# Supplementary material for: Augmented ultrasonography with implanted CMOS electronic motes
Source: Nat Commun. 2022 Jun 20;13:3521. doi: 10.1038/s41467-022-31166-x (PMC9209459; doi:10.1038/s41467-022-31166-x)
Supplement: Supplementary file 1 — Supplementary Information [file 41467_2022_31166_MOESM1_ESM.pdf]

Supplementary Information

Augmented ultrasonography with implanted CMOS electronic motes

Yihan Zhang[1, 2], Prashant Muthuraman[1], Victoria Andino-Pavlovsky[1], Ilke Uguz[1], Jeffrey Elloian[1], Kenneth L. Shepard[1]

1. Department of Electrical Engineering, Columbia University, New York, NY 10027

2. School of Integrated Circuits, Peking University, Beijing, P. R. China

1. Input impedance of the piezoelectric transducer

For a piezoelectric transducer, changing the electrical impedance loading the element affects the acoustic impedance. In a similar manner, the mechanical loading affects the electrical impedance. The measured electrical impedance of the 1-mm-by-1-mm-by-0.5-mm lead zirconate titanate (PZT-5A, Piezo Systems) used here is shown in Fig. S1a, where the transducer is mounted on the mote's FR4-based printed circuit board (PCB) submerged in water. The equivalent circuit for this transducer is shown in Fig. S1b. Previous work[4] has shown that with air-backed piezoelectric crystals, a robust inductive band forms between the series and parallel resonant frequencies over a span of approximately 100 kHz for typical element values. However, only a weak resonance is observed here without an inductive band, which is likely due to the lack of proper mechanical boundary conditions that are required for a high-quality-factor mechanical resonance.

Producing such a resonance necessarily complicates the mechanical design of the mote, leading to a higher implant volume. To minimize the overall volume, we instead design the mote to the capacitive impedance response, on the order of 10s of pF for the transducer employed here, which dominates the rest of the impedance spectrum. In this case, the circuit interface to the transducer functions over a much wider frequency range, and smaller transducer sizes can be easily accommodated without changes to the mote circuitry. Fig. S1b shows the capacitive model fit to the impedance data.

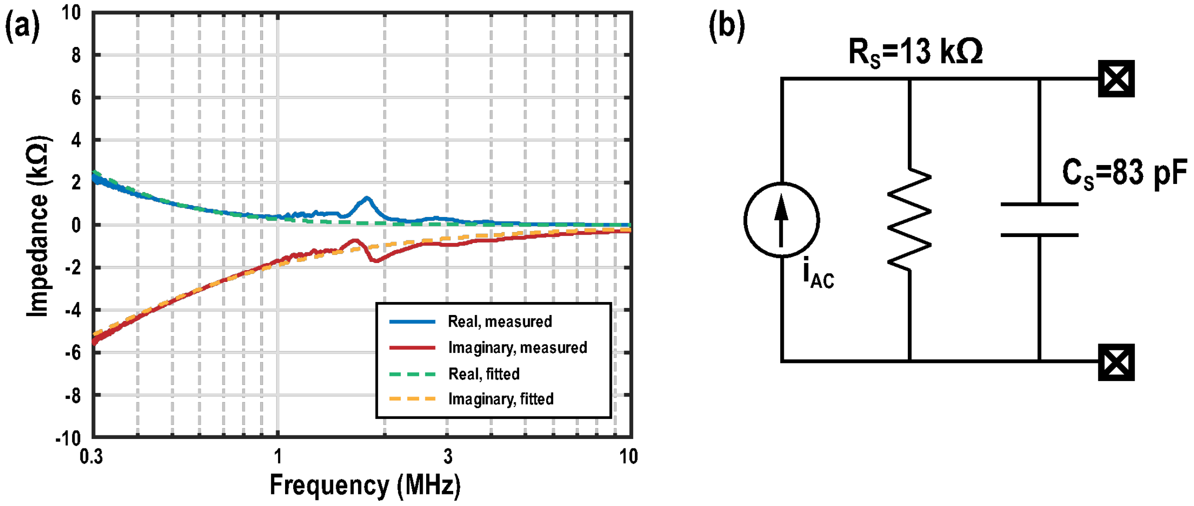

Figure S1. Input impedance of the piezo element. (a) measured input impedance (real part and imaginary part) of a 1 mm (W) by 1 mm (L) by 0.5 mm (H) lead zirconate titanate mounted on FR4-based PCB submerged in DI water, together with model fit, and (b) the equivalent circuit model producing the fitted curve.

2. Switch-only rectifier

When the input impedance is dominated by a capacitance on the order of 10s of pF, an inductor value of approximately 100 μH would be required for conjugate matching at a 4-MHz center frequency. Even with bulk ferrites, the smallest such inductor available to date is still in a 0805 package, reducing significantly the ability to scale the total mote volume. To harvest power from

the piezoelectric transducers without such inductors, we instead choose to translate one of the techniques widely used in sub-kHz mechanical energy harvesters, the “switch-only” rectifier topology[23], to the MHz frequency range.

A switch-only rectifier achieves a higher efficiency than simple active rectifiers by increasing the conduction angle when connected to sources whose impedance is dominated by a capacitive component. Fig. S2 illustrates the principle of the switch-only operation, where the transducer is modelled as a capacitor (C_S) in series with a current source. With an ideal rectifier, the power from the piezo crystal only flows into the storage capacitor when the voltage across the transducer (V_{PZ}) is equal to V_{CC}, the already stored voltage on the load capacitance, C_L. At the end of the conduction phase, the transducer needs to slowly discharge C_S, and the next conduction phase begins when V_{PZ+} is less than $-V_{CC}$. The switch-only topology adds a switch across the transducer, as well as a pulse generator, shown in blue in Fig. S2. The active diode now is cascaded to the full bridge rectifier, and the output of the comparator from the active diode is used to track the conduction phase. After one conduction phase ends, the switch closes for a short period of time, zeroing the voltage across C_S. This effectively shortens the discharge time required to reach the next conduction phase, increases the total conduction time. A theoretical maximum efficiency boost of 100% is predicted[23], assuming ideal diodes and switches are used to implement such a rectifier. In practice, we simulated an efficiency boost of approximately 46% at the designed load level by the use of this topology compared to optimized passive rectifier-based approaches[12,22].

Using our measured piezo model and assuming a load resistance of 2.72 GΩ in parallel with a decoupling capacitor, simulation shows the active rectifier can support 1.95 nW of harvested power from a 50-ppm-duty-cycle, 225-kPa-ultrasound pressure source. At this point, the majority of the power is dissipated in voltage clamp (see Fig. S2) within the active rectifier. A load

resistance of 180 MΩ corresponds to maximum power transfer; in this case, the active rectifier can harvest up to 8.59 nW.

The 225-kPa-pressure source delivers an incident acoustic power of 835 nW. Acoustic impedance mismatch between soft tissue (~1.54 MRayls) and the piezo crystal (~36 MRayls) reflects 84.3% of this acoustic power, leaving 131 nW for harvesting. Part of this acoustic power is lost in the mechanical to electrical power conversion, given by η_P, the piezoelectric efficiency; additional loss comes from the electrical impedance mismatch between the piezo crystal and the rectifier, given by η_M, the matching efficiency. An air pocket in the package providing backing to the piezo transducer increases η_P could be increased by the addition of an air pocket in the package, backing the piezo transducer. Resonant power harvesting can boost η_M. However, both of these techniques have negative attributes. If an air pocket is included in the mote, its area needs to be the same as that of the piezo, with a thickness beyond one wavelength. In our case, this requires an added volume on the order of 1 mm × 1 mm × 0.4 mm. The addition of an inductor for resonant power harvesting usually leads to bulky inductors on package. For the values required here, the inductor will be at least be of the scale of 0805 packaging, leading to an extra 2 mm × 1.2 mm × 1.2 mm in volume. Off-resonance switch-only based power harvesting only requires an on-chip switch, with an area cost in the order of 10s of μm^2 (W/L = 10 µm/0.6 µm transistor used in this work). An off-resonance power harvesting scheme also allows the mote to adopt to a wider range of carrier frequencies.

The interfacing circuits uses thick oxide IO devices for higher voltage compliance. A voltage clamp is added here to clamp V_{CC} to 1.2 V, such that the rectified voltage can be safely used to operate thin oxide (1.8 V core) devices.

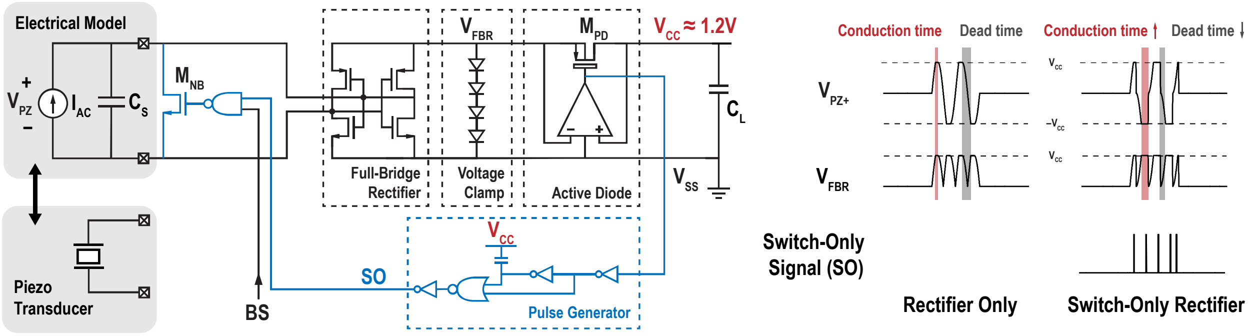

Figure S2. Simulation results comparing a traditional rectifier and a switch-only rectifier with the switch-only-operation-related circuitry highlighted in blue.

3. Decision logic for data downlink

The decision logic determines whether the downlink data is a '0' or '1' based on the pulse width received at the mote. Since all pulses in the same pulse packet have the same pulse width, every pulse can be used for downlink recovery. A four-bit `count` signal indicates the pulse width as the number of half cycles seen at the output of the full bridge rectifier (V_{FBR}) for the pulse that triggers the clock synchronization circuit. The decision logic, in turn, sends this `downlink_data` as well as an indicator that the downlink data is ready, `data_ready`, to the higher layers of the protocol stack. A pseudocode description of the decision logic is available as Table S1. To determine `downlink_data`, the decision logic monitors the historical maximum observed "count" value (`value_max`). Once `count` in one frame becomes smaller than `value_max` by three, `value_threshold` is then updated for future data decisions, `downlink_data` is set to 0 if the incoming `count` value is less than `value_threshold`, and 1 otherwise, and `data_ready` is asserted. In addition, if `downlink_data` has the same value for more than 32 consecutive frames, the decision logic resets itself, such that a new `value_max,` and thus a new `value_threshold` can be generated. This "confusion" feature helps to avoid the possibility that `value_max` is set to

an incorrectly high value as a result of bit errors. The link layer protocol uses “0101” as the header to ensure that normal operation will never produce this “confusion” state.

Table S1. Pseudocode for the decision logic in data downlink

```
module decision_logic
    input:  reset, clock, count
    output: downlink_data, data_ready
    registers:  value_threshold, value_max, data_prev, confusion_counter
    parameters: confusion_limit = 32
    assign downlink_data = !(count < value_threshold)
    always @ (posedge clock):
        if (reset): reset all registers and outputs to 0
        else:
            data_prev <= downlink_data
            if (count > value_max): value_max <= count
            if (count < value_max - 2):
                value_threshold <= count + 3
                data_ready <= 1
            if (data_prev == downlink_data):
                confusion_counter += 1
                if (confusion_counter == confusion_limit):
                    reset all outputs and registers to 0
            else: confusion_counter <= 0
```

4. Backscatter modulator

For data uplink, on-off keying of the electrical impedance of the transducer is used to produce a backscatter signal. In Fig. 3f, when the uplink data is 1, we open the NMOS switch M_{NB}, while when the uplink data is 0, M_{NB} is on and shorts the transducer for a maximized data modulation depth. When the transducer is shorted, no power, clock, or down link data can be recovered. Furthermore, since the generation of the uplink data bit is fully synchronized to the recovered clock, whenever a 0 is sent, the recovered clock is stalled, as M_{NB} remains in the short state, deadlocking the chip. An asynchronous approach is required in the backscatter modulator to prevent this.

The backscatter modulator consists of two matched pulse-generation paths, each consisting of five-bit-tunable, falling-edge-sensitive delay elements as shown in Fig. 3f. Fig. S3 shows one of these pulse generation paths. A falling edge at the input generates an inverted (1-0-1) pulse whose

pulse width is set by the delay element. Backscatter modulation logic, the pseudocode of which is given in Table S2, acts as the interface between the synchronous signal `uplink_data` and the asynchronous pulse generation paths. To ensure robust operation in the case of the transmission of multiple 0s, the logic block generate two signals, `data_a` (for auxiliary) and `data_m` (for main). In the absence of multiple 0s, `data_m` is the same as `uplink_data` and `data_a` stays high. When a second 0 is to be sent, `data_a` is pulled low while `data_m`, at a low level from the previously sent bit 0, resets to 1. With this implementation, each uplink bit 0 takes two clock cycles, or two frames, to transmit. This results from the fact that the `bs` signal resetting to high asynchronously triggers the clock recovery, which only occurs in the next frame. With such an implementation, transmitting a single bit 0 now takes two clock cycles. To keep a constant data rate in the uplink channel, the backscatter modulator logic artificially delays every bit 1 to also take two clock cycles. As a result, the overall uplink data rate is half of the frame rate.

Another required function of the backscatter modulator logic is to find a working delay value on start-up such that the pulse width for bit-0 transmission is as close to one clock cycle as possible. Upon power-on reset, signal `en` is set to 0, which gates off uplink data transmission to allow perfect clock recovery. The backscatter modulator monitors the signal `bm` and tunes the digital delay value supplied into the delay elements, until a working delay value is found. Finally, signal `en` is set to 1, enabling uplink data transmission.

Table S2. Pseudocode for the backscatter modulator logic in data uplink

```
module backscatter_modulator_logic
    input:  reset, clock, uplink_data, bm
    output: data_m, data_a, delay, en
    states: TUNE, READY
    if (reset): delay <= 31, en <= 0, data_m <= 1, data_a <= 1 state = TUNE
    else:
        case TUNE:
            data_m <= 0,
            @ next negedge clock:
```

```
            if (bm == 0): confirm at off state
            @ next posedge clock:
            if (bm == 1): confirm at on state
            if (confirmed at off and on state): en <= 1, state <= READY
            else: delay -= 1, data_m <= 1
        case READY:
            always @ (posedge clock):
                if (uplink_data == 0):
                    if (data_m == 0): data_m <= 1, data_a <= 0
                    else: data_m <= 0, data_a <= 1
                else:
                    data_m <= 1, data_a <= 1, wait for one clock cycle
```

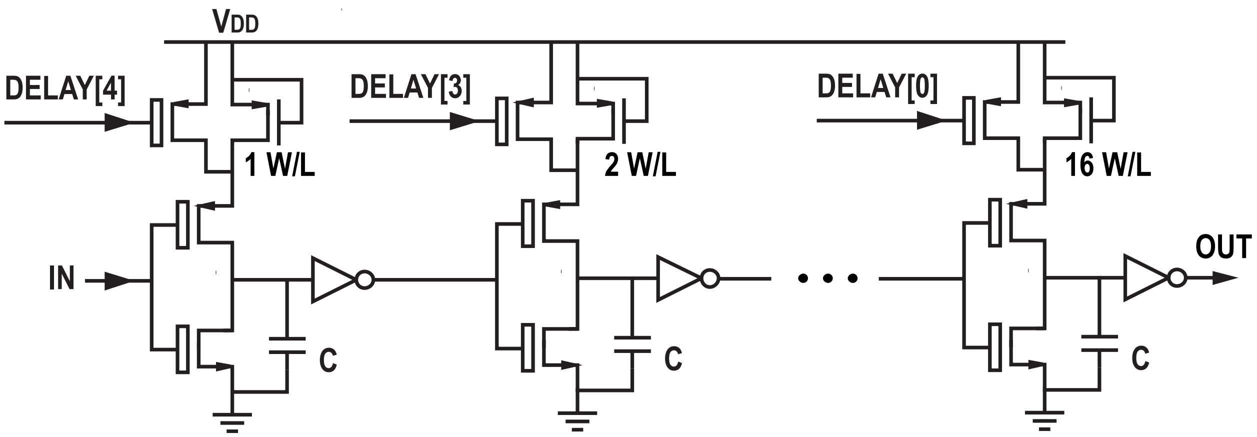

Figure S3. The schematic of the five-bit-tunable, falling-edge-sensitive delay elements.

5. Link layer and application layer

The link layer frame structure is shown in Table S3. `INST` in the table refers to the four-bit instruction code in the application layer; `ARG` stands for the eight-bit argument for each instruction code; while `RET` represents the return value from the mote.

Table S3. Link layer frame structure

**Frame structure for data downlink**																
Bit	15	14	13	12	11	10	9	8	7	6	5	4	3	2	1	0
Field	0101				INST				ARG							
**Frame structure for data uplink**																
Bit	15	14	13	12	11	10	9	8	7	6	5	4	3	2	1	0
Field	00		INST				RET								P	P’

Table S4. Application layer instructions

Instruction (INST)	Code	Argument (ARG)	Return Value (RET)
QUERY_ID	0101	Do not care	ID
HELLO	0110	ID	HELLO (0101 0101)
CONFIG	1010	ID	ACK (1010 1010)
STORE	1001	DATA	ACK (1010 1010)
LOAD	1100	ID	DATA
HP_MODE	1101	ID	ACK (1010 1010)

6. Custom Delay-and-Sum Algorithm

To detect the backscattered data in the reconstructed image, a custom delay-and sum algorithm is necessary to generate floating-point precision images, as the hardware accelerated version implemented in the Verasonics Vantage 256 (Verasonics Inc.) system rounds the reconstructed image to eight-bit fixed-point values for display purposes. A custom implementation here effectively reduces the unnecessary quantization noise, significantly increasing the SNR for the uplink data.

The delay-and-sum algorithm is briefly outlined as follows. For each focused ultrasound beam generated (or the transmit event), the echo as a function of time is recorded at 15.625 MSamples/s for 1280 samples, using up to 127 elements around the x coordinate (see Figure 2 for the coordinate system) of the focal point unless the beam is too close to the edge of the transducer array. The focal depth of the beam in the transmit event, as well as the x-coordinate of each receive element, are then used to calculate the sound wave's travel distance from the corresponding receive element to the focal point of interest. This travel distance is converted to time delay (assuming a uniform sound speed of 1540 m/s in the media) and further quantized into the number of samples. The received waveform from all 127 element in one transmit event is then delayed by their

corresponding number of samples, and then summed together to generate a 1-by-1280 vector as the result. The final B-mode image is then generated by combining the results from all 192 transmit events, which is subject to further filtering and processing as described in Methods.

For cases in which devices at multiple depths work in parallel, compound reconstruction is performed by first running delay-and-sum multiple times, assuming a focus at the depths of interest (or receive foci), instead of that in the transmit event, and generating multiple B-mode images. These B-mode images are then combined using four-mm-long rising cosine tapers after envelope detection.

7. Electrical performance of the chip operating with imaging ultrasound

To fully verify the functionality of the designed integrated circuit with imaging ultrasound, the chip is packaged with the piezoelectric transducer and wirebonded for on-chip logic signal probing. To verify the chip's performance under realistic ultrasonography situations, B-mode ultrasound pulses are generated using the Verasonics Vantage 256 research ultrasound system with a L12-3V linear array probe (Verasonics Inc.). For the ultrasound imaging session, each frame consists of 192 spatially separate scan lines, or ray lines, with a 100-μs delay between adjacent scan lines. For each scan frame, either a three-cycle-long (0.75 μs) pulse, or a five-cycle-long (1.25 μs) pulse to represent downlink bits 0 and 1, respectively. Fig. S4 shows the hydrophone (Onda Cooperation) measurement of the emitted focused pulse. Nonlinearities in the linear array transducer and its driving waveform are responsible for the "tail" observed in the pulse in Fig. S4.

Fig. S5 shows the monitored on-chip supplies during start up when placed within the field of view of the imaging system. The complete start up process takes about 4.7 seconds. When a "QUERY_ID" instruction (with ARG = 0101 0101) is sent from the linear array transducer, the

mote correctly recovers the downlink data and delivers the correct uplink frame fully synchronized to the frame rate (Fig. S6).

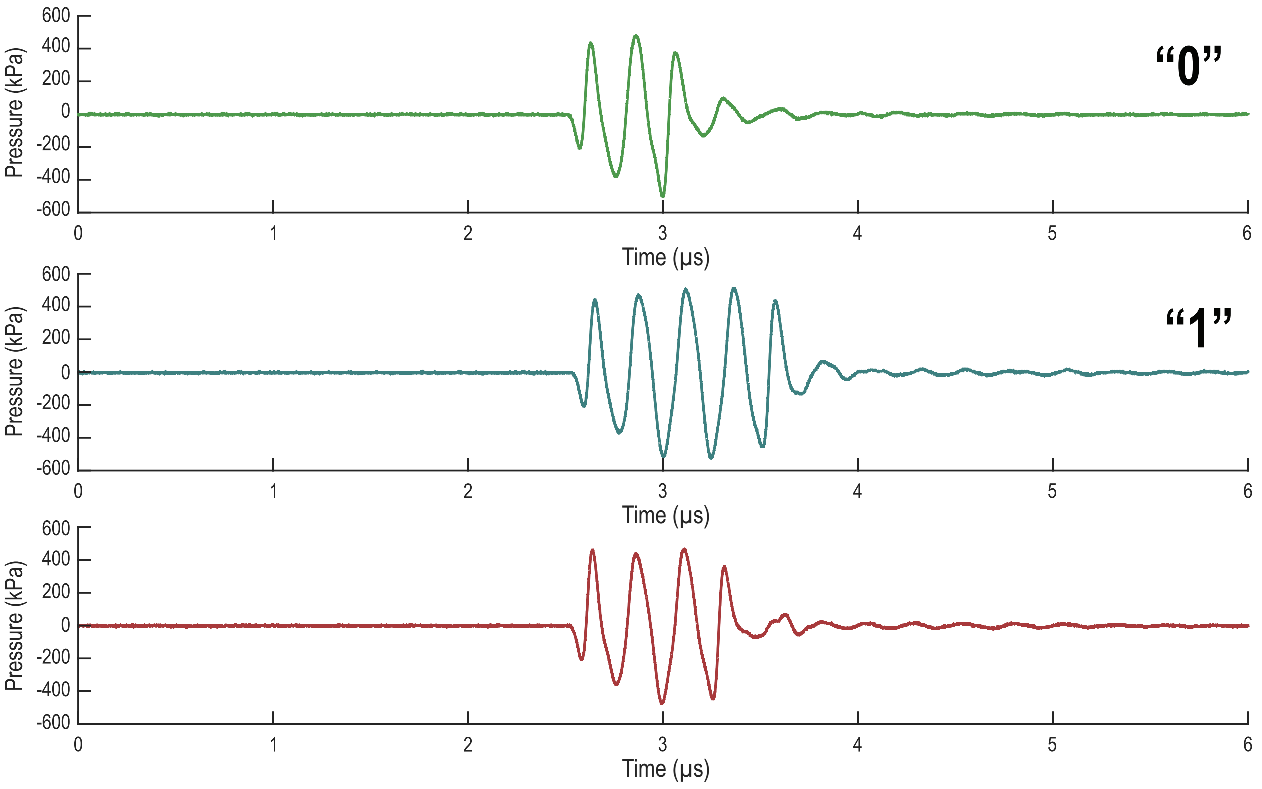

Figure S4. Measured ultrasound waveforms using a hydrophone and an oscilloscope for three-cycle, five-cycle, and four-cycle ultrasound pulses, respectively.

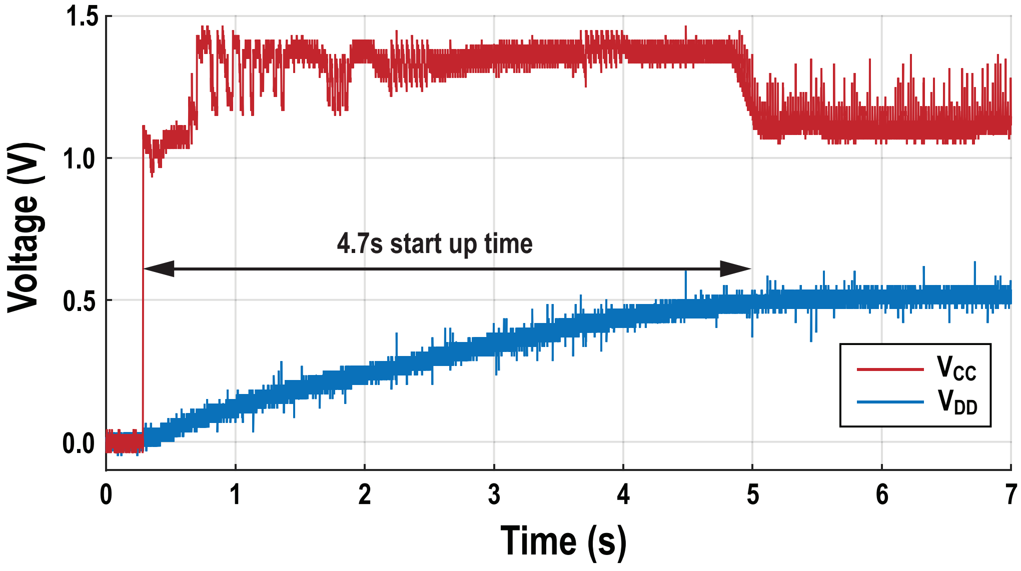

Figure S5. Recorded V_{CC} and V_{DD} supply line voltage waveforms after the start of the ultrasound imaging.

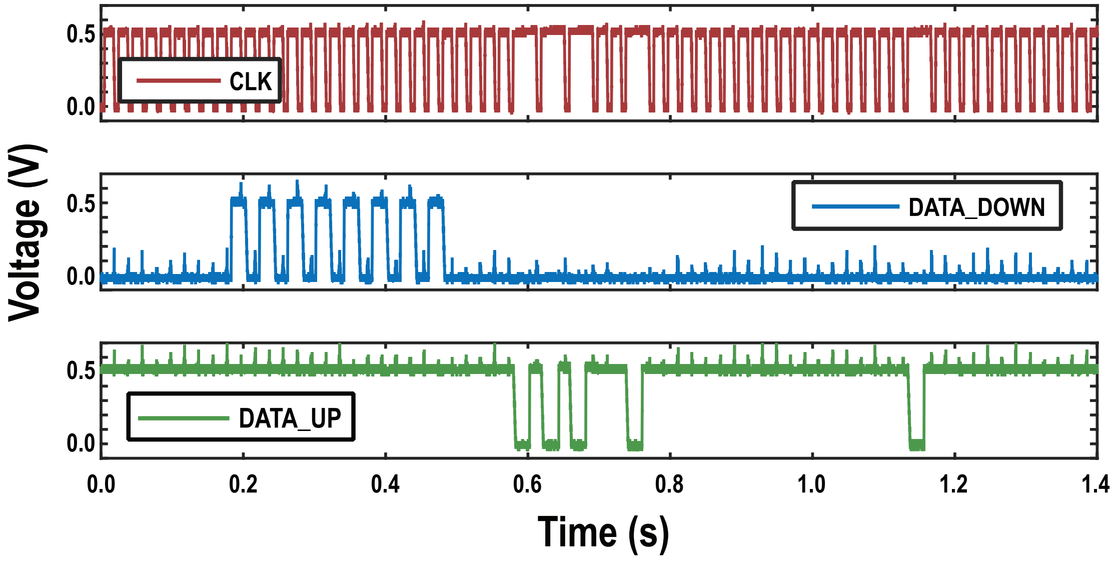

Figure S6. Clock, downlink data, and uplink data waveforms when a linear array transducer broadcasts the instruction "QUERY_ID".

7. Measured backscatter modulation from the mote

To maximize the backscatter modulation depth, M_{NB} switches between on and off, changing the effective electrical load on the piezo element. To capture the electrical changes, I-V curves are simulated assuming the core circuits are properly operating under a 1.2 V V_{CC}, at states in

which M_{NB} is either on or off. This simulation result is plotted as Figure S7. The equivalent resistance change is between 1.26 GΩ in the off-state and 364 Ω in the on-state.

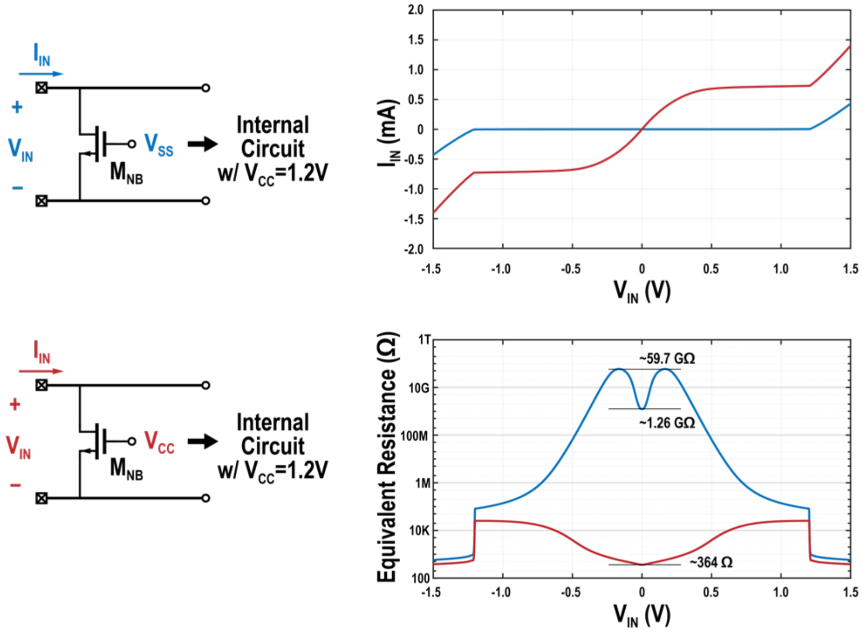

Figure S7. Simulated I-V and derived equivalent electrical resistance of the mote as seen from the piezo crystal with M_{NB} being either on or off.

This loading resistance change on the piezo crystal creates an effective acoustic impedance change, which further induces a backscatter amplitude shift when digital data is sent to the gate of M_{NB}. This change in the amplitude of the backscatter ultrasound can be captured by the Verasonics system. Two recorded scanlines with different uplink data at the same lateral (x-direction) location are compared in Figure S8. Here a difference of 19% in backscatter amplitude at the axial (z-direction) location of the mote is observed.

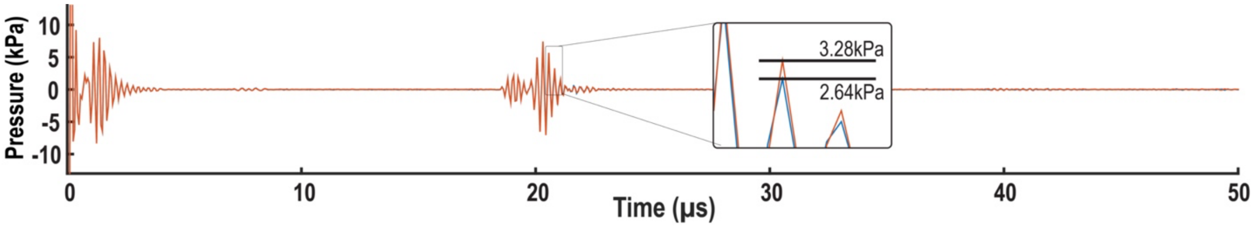

Figure S8. Recorded scanlines at the same lateral (x-direction) location when the uplink data bit is different; the red line showing the case when the uplink data bit is “1” (M_{NB} is open) and the blue line showing the case when the uplink data bit is “0” (M_{NB} is closed).

8. Image quality degradation in the vicinity of the mote

Since the mote is a strong reflector of ultrasound, images suffer from reduced imaging quality for areas around and below the mote. In the lateral direction, a finite beam width creates an image of the mote larger than its actual width. In the axial direction, the mote blocks the ultrasound energy, dimming the region in the B-mode image below its location. In addition, multiple reflections leads to copies of the mote (reverberant artefacts). The exact amount of interference in the image depends on various parameters, including the beam profile, the dimension of the mote, and the relative position between the mote and the transducer array. For example, side lobes can create horizontal phantom images of the mote if they are strong enough, while a wide main beam convolutes with the mote’s dimension, leading to a spread in the width of the mote’s image. In the case that the mote is smaller than the width of the beam (either in the axial direction or in the elevational direction), then it does not completely block ultrasound energy, leading to a reduced shadowing of the region below.

To quantify the level of “shadowing” caused by the use of the mote to the image, 3D k-Wave simulations are performed here using ultrasound transducer setup similar to the L12-3V linear array probe deployed in the experiments. Spherical regions of randomized scatterers are added to mimic a realistic tissue environment, and offer intuitive comparison of the image quality. The mote is modeled here by a 1.0 mm × 1.0 mm × 0.5 mm PZT crystal. Within the region of simulation, a mote is either absent for the baseline result, or placed at 10 mm, 15 mm, 20 mm and 25 mm apart from the source transducer array. The focus of the beam is set to 15 mm for all simulations. These setups together help characterize the effect from varying beam width in the axial direction. The

results of these simulation are summarized in Figure S9, which shows both reconstructed B-mode images for an intuitive evaluation and the recorded maximum pressure in space as a quantitative evaluation of shadowing.

From the resulting B-mode images, the image of the mote shows minimum lateral spread at the focal depth, while showing up to ±2 mm of spread at 25-mm depth. Reverberant artefacts are no longer present 3 mm below the mote's location. In the pressure recording, the placement of the mote leads to pressure attenuation that shadows the regions below it, as expected; however, this shadowing effect quickly goes away for deeper regions. At approximately 5 mm beneath the mote, the pressure reduction is no longer significant. This shadowing effect is also reduced when the mote is placed in the near field, before constructive interference happens.

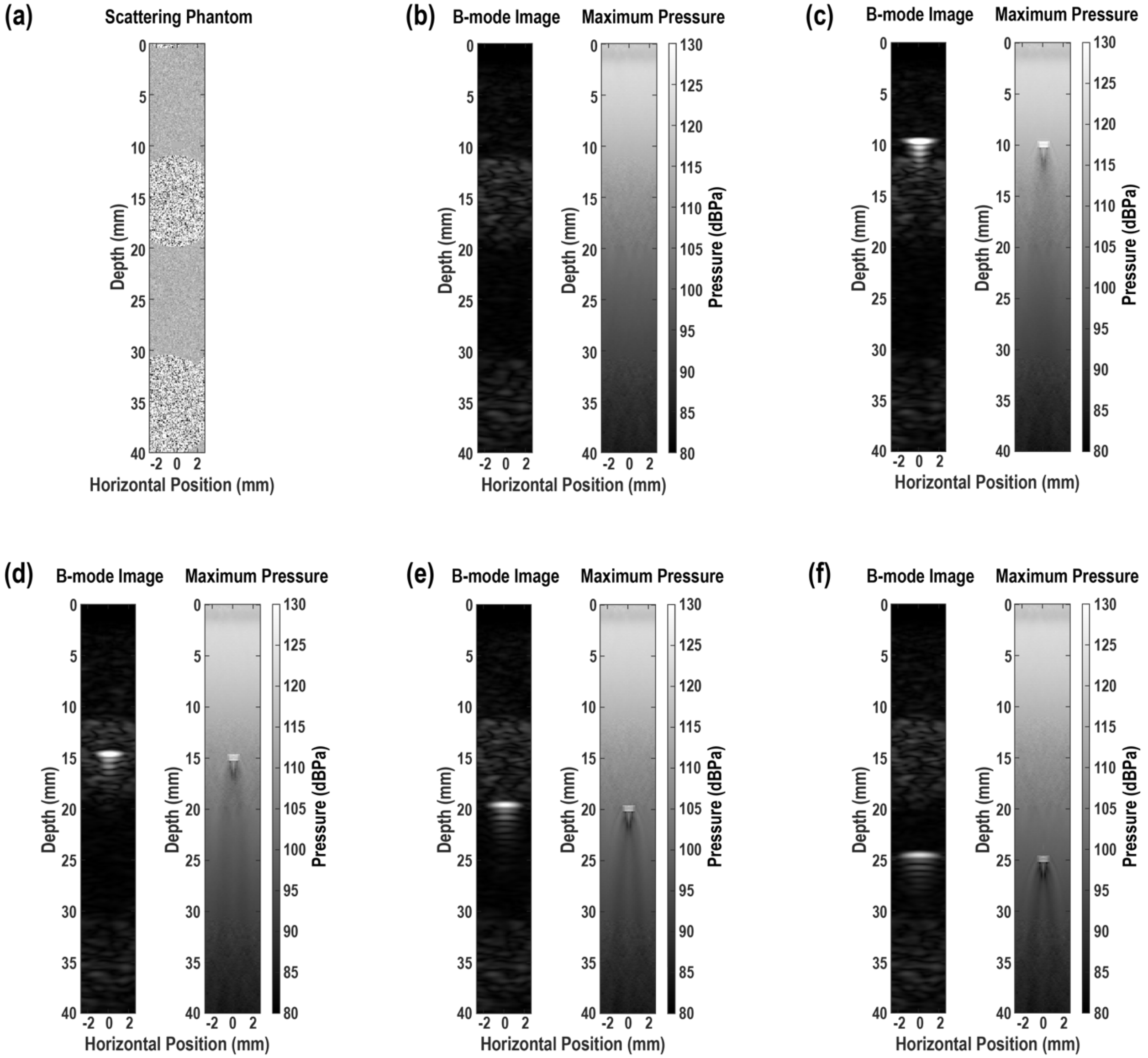

Figure S9. Phantom setup (2D cross section at zero elevational distance, i.e., y = 0) and 3D ultrasound simulation that estimates the amount of ultrasound energy blocking by the placement of the mote; (a) medium set up with regions of random scatterers; (b) simulation results for reconstructed B-mode image and maximum pressure distribution with no mote (baseline), with the mote placed at (c) 10-mm, (d) 15-mm, (e) 20-mm, and (f) 25-mm distances from the transducer.

9. Proximity effects from closely placed motes

The operating principle of this augmented ultrasonography allows multiple motes placed within the same field-of-view; however, when they are placed in proximity, data interference may happen that makes them less distinguishable from each other. This is because data from two motes

placed within the width of a scanline can get captured and reconstructed into a single point at the resulting B-mode image. Here, a setup shown in Figure S10 is used, with 13 mm thick chicken meat on top of two motes placed with a separation of about 3.3 mm with the imaging transducer array placed 34 mm above the motes. Data signatures captured show spatial separation that is clearly distinguishable with SNRs of 34 dB (left) and 25.9 dB (right).

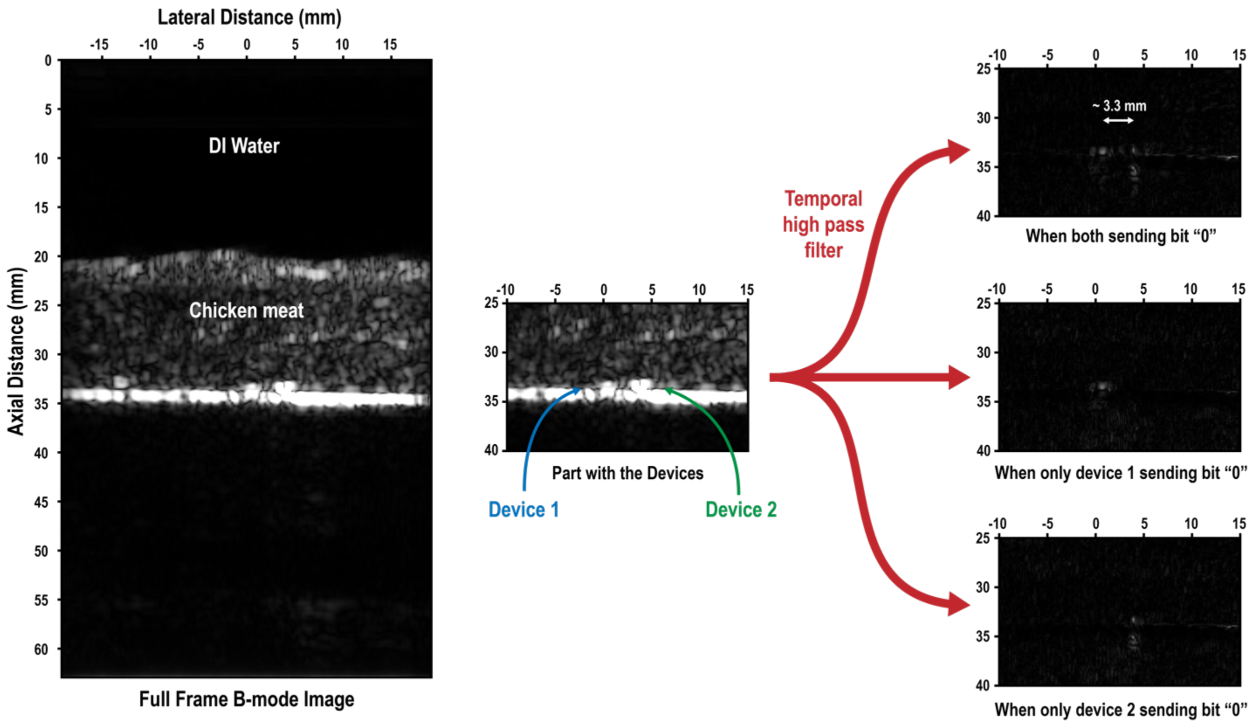

Figure S10. Experiment verifying the ability to distinguish motes places3.3-mm apart at a distance of 34 mm from the source transducer in an *in-vitro* setup with spatially separate data signatures clearly distinguishable in the reconstructed B-mode image.
