# Supplementary material for: Augmented ultrasonography with implanted CMOS electronic motes
Source: Nat Commun. 2022 Jun 20;13:3521. doi: 10.1038/s41467-022-31166-x (PMC9209459; doi:10.1038/s41467-022-31166-x)
Supplement: Supplementary file 2 — Description of Additional Supplementary Files [file 41467_2022_31166_MOESM2_ESM.pdf]

Title: Supplementary Movie 1
Description: Two devices' backscattered data, captured within the field of view of a single linear array transducer in a tissue phantom.

Title: Supplementary Movie 2
Description: The device's operation *in vivo*.
